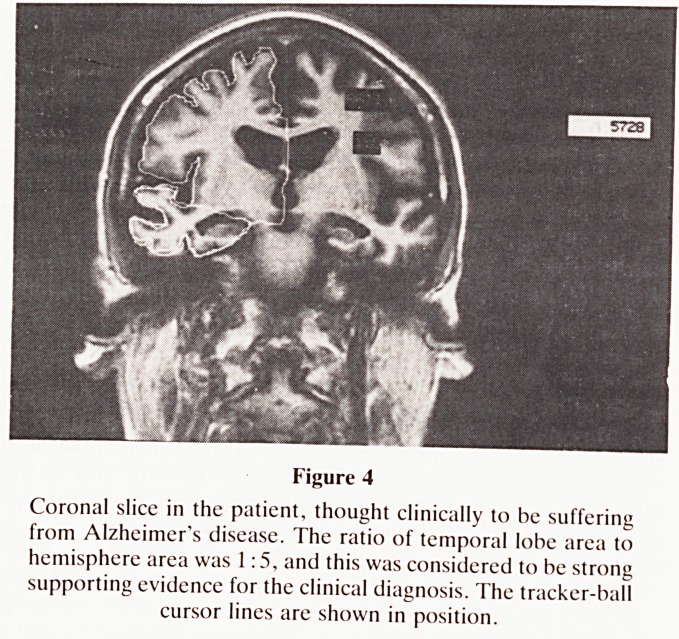# M.R.I. in Alzheimer's Disease
*Based on a paper read at the Plymouth meeting of the British Association of Neuroradiologists, October 1989.


**Published:** 1990-09

**Authors:** J. L. G. Thomson

**Affiliations:** Consultant Radiologist, Frenchay Hospital, Bristol

## Abstract

At present, the clinical diagnosis of Alzheimer's disease is shown to be unreliable. In the absence of any laboratory or blood test, a study of the brain configuration using Magnetic Resonance Imaging, can help to demonstrate the differential atrophy involving the temporal lobe more than elsewhere, which is present in the majority of cases of Alzheimer's disease. Area measurements have been obtained in the coronal slice and comparison made between 20 controls and 10 patients suffering from early dementia.


West of England Medical Journal Volume 105(iii) September 1990

### M.R.I. in Alzheimer's Disease*

**J. L. G. Thomson, MD, FRCP, FRCR**
Consultant Radiologist, Frenchay Hospital, Bristol

* Based on a paper read at the Plymouth meeting of the British Association of Neuroradiologists, October 1989.

### ABSTRACT
At present, the clinical diagnosis of Alzheimer's disease is shown to be unreliable. In the absence of any laboratory or blood test, a study of the brain configuration using Magnetic Resonance Imaging, can help to demonstrate the differential atrophy involving the temporal lobe more than elsewhere, which is present in the majority of cases of Alzheimer's disease. Area measurements have been obtained in the coronal slice and comparison made between 20 controls and 10 patients suffering from early dementia.

### INTRODUCTION
Dementia is a common disorder, and is increasing in incidence and importance as the population ages. Its present frequency is assessed at different levels, depending on the reference, e.g. one in six over the age of 65 years[1], and one in twenty by the age of 70 years[2]. It has been calculated that in a U.K. Health District of one quarter of a million people at the present time, 3,000 elderly dements will need care[3]. It is said that Alzheimer's disease is the most common cause of dementia in the elderly, and in the U.S.A. it is said to be the fifth most common cause of death, but it is also said that only 13% of patients dying in the U.S.A. have a necropsy. It is recognised that it is a very difficult disease to diagnose clinically, and it is only by microscopy, either by biopsy or necropsy, that Alzheimer's disease can be confidently confirmed. Alzheimer was a German professor of Psychiatry, with a particular interest in microscopy, and the diagnosis of the disease named after him rests on neuronal loss, the presence of neurofibrillary tangles and plaques in the cerebral cortex. Nowadays it is accepted that neurofibrillary tangles may occur in the normal aging brain, in the hippocampal pyramidal layer and the entorhinal cortex, but their presence in the six-layered neocortex around the lateral part of the para-hippocampal gyrus with spread laterally over the adjacent cerebral hemisphere, is diagnostic of Alzheimer's disease[4]. The presence of senile plaques, which are collections of swollen abnormal axons and dendrites, may also occur in the normal aging neocortex, but alone they are not pathognomonic of Alzheimer's disease[4]. A microscopic section is shown in Fig. 1.

A short list of differential causes of dementia is shown in Table 1, and particular emphasis should be drawn to senescent forgetfulness and depression, two common causes of early memory loss, which is often the first symptom of Alzheimer's disease. In one series (Homer *et al.* 1988)[5], patients presenting with dementia were examined extensively by clinical tests, but of 13 cases diagnosed in life as Alzheimer's disease, only six were confirmed at neuropathological post-mortem examination. Considerable research is at present being undertaken for an effective treatment of this disease[6], and it is therefore important for this and other reasons that the diagnosis is assured prior to administration of the drug[7]. Unfortunately no laboratory test is available. A simple skin test was reported in the Press from the U.S.A. but evidently the reliability is in doubt[8]. Some observations have been reported of changes in the hippocampus as shown by MRI and demonstrated in the sagittal plane[9], but inasmuch as the whole temporal lobe shows striking shrinkage in the coronal plane at post-mortem, as shown in the section illustrated (Fig. 2), a study of the coronal slice of the whole brain at MRI in life, seemed a potentially helpful method for diagnosing the condition.

### METHOD
Using a Picker MRI Scanner 2055HP, multisection coronal slices 5 mm thick, perpendicular to the long axis of the temporal horn, were taken through the heads of 20 controls, using an Inversion Recovery Sequence (IR 1500/500/30). The slice through the anterior aspect of the temporal horn and the hippocampus was used for the study (Fig. 3). The 20 controls were collected from healthy volunteers, and a few patients with minimal symptoms, considered non-organic, with scans adjudged by two radiologists to be normal. Area measurements using the in-built computer and the tracker-ball cursor were obtained so that the ratio of the size of the temporal lobe to the whole hemisphere could be calculated. The mean ratio of the temporal lobe to the whole hemisphere could be calculated. The mean ratio of the temporal lobe area: hemisphere area in these 20 controls was found to be 1:3.13, with a variation from 1:2.95 to 1:3.48. Ten patients with early memory loss and mild dementia, yet still co-operative enough to lie still in the MRI Scanner, and thought to be suffering from early Alzheimer's disease, were examined in a similar way. The ratios of temporal lobe area to hemisphere area was higher than the control group in seven cases, with a top ratio of 1:5. The latter figure indicated severe temporal lobe atrophy. In contrast, two further cases showed a ratio within the normal range, and in all other ways the scans of these two were considered normal. The tenth patient had dilated ventricles with normal to small sulci, an appearance thought to raise the possibility of a low pressure hydrocephalus (L.P.H), but no further tests were done. No coronal views were obtained in this patient. The coronal view of the patient with a 1:5 ratio is illustrated in Fig. 4, with the tracker-cursor lines in position for the area measurements. The lateral and third ventricles are also dilated, and well seen in this view. It was considered from this slice that the MRI evidence supported the clinical diagnosis of Alzheimer's disease. In the others with less impressive ratio figures, the MRI findings were considered still to support the clinical diagnosis of Alzheimer's disease, but with a correspondingly lesser degree of emphasis. The two patients with normal scans were thought to require further clinical assessment as Alzheimer's disease appeared very unlikely. Finally in one of the patients found to have evidence of a small temporal lobe, and with an abnormal ratio, numerous high intensity shadows were present on the T2 axial views, alongside the lateral ventricles, indicative of small vessel ischaemia, so that the MRI diagnosis was one of arterio-sclerotic encephalopathy combined with Alzheimer's disease. This combination of two diseases is a well recognised occurrence in dementia, and was the only case in our series of ten with early memory loss.

### DISCUSSION
Hubbard *et al.* (1981) carried out a quantitative study of cerebral atrophy in old age and senile dementia by comparing brain volume and cranial capacity at necropsy, and they showed that in Alzheimer's disease, the temporal lobe is more severely affected than other parts of the brain, even when global atrophy is severe, although in some dements naked eye changes can be minimal[10]. It is also recognised that pathological ventricular enlargement is by no means a reliable marker for dementia, and there seems no useful correlation between temporal lobe shrinkage and the size of the ventricular system[11].

In assessing the value of CT in dementia, Bradshaw *et al.* (1983) investigated 500 consecutive patients presenting with dementia, and found that 82 had a normal scan, and more than 10% had treatable lesions[12]. The importance of accurate diagnosis in cases of dementia was stressed, and it was suggested that patients demented for more than a month and less than a year should have a brain scan. With CT however the plane of the slice is traditionally axial, and a coronal slice usually requires that the patient lies prone, or that the original data from the axial slices be reformatted. With MRI however, with its facility to examine a patient in any plane, whilst the patient lies supine, and with a multislice technique, it is simple to examine a brain in axial and coronal planes, and any other as required, providing the patient is co-operative enough to lie still for the examination to take place. For those with early memory loss, this is usually no problem, and the greatest difficulty in the U.K. may be to find time on an MRI machine, such is the poor provision of the equipment at present. At the Bristol MRI Centre we use a multiecho axial sequence (1800/30/100), and inversion recovery coronal sequences, the former particularly to check the size and position of the ventricles, and to check for other lesions, and the latter to confirm lesions already shown, and to allow comparison of the temporal lobe size in relation to the whole hemisphere, if Alzheimer's disease is queried clinically. The better soft tissue contrast provided by MRI, which allows clearer demonstration of other lesions, such as ischaemic areas, is another advantage over CT, and furthermore the artefactual shadows that occur with bone or dental amalgam at CT, do not occur with MRI.

However, it has to be recognised that the measurements and the ratios as described above have been carried out on only a very few patients, and in none of these have biopsy or necropsy confirmation of Alzheimer's disease or otherwise been obtained. Should the opportunity present, a larger series of cases and neuropathological microscopy for confirmation, should clearly be valuable in assessing reliability of such a test. One has to remember too that in the early stages of the disease when symptoms are minimal, the changes in the brain are also likely to be minimal. It is possible moreover that as well as the ratio of temporal lobe size in relation to hemisphere size, further help may be obtained by sagittal slices for closer study of the hippocampus. But until a simple laboratory or skin test become available for Alzheimer's disease, MRI examination could have a valuable role to play, and further use of this simple test would seem to be worthwhile.

### REFERENCES

1. TERRY, R. D. (1976) Dementia: a brief and selective review. *Arch. Neurol.* **33**, 1–4.
2. ROTH, M. (1978) Epidermological Studies. In Katzman, R., Terry, R. D., Bick, K. L., eds. Alzheimer's disease: senile dementia and related disorders. New York: Raven Press 1978: 337.
3. College Committee on Geriatrics of the Royal College of Physicians (1981) Organic mental impairment in the elderly. *Jr. Coll. Physicians London*, **15**, 141–67.
4. MOSS, T. H. (1990) Neuropathology Dept. Frenchay Hospital. Personal Communication.
5. HOMER, ANN C., HONAVAR, M., LANTOS, P. L. *et al.* (1988) Diagnosing Dementia: do we get it right? *Brit. Med. Journ.* Vol. 297, Oct 8, 894–896.
6. Drug and Therapeutics Bulletin (1990) Vol 28, no. 11, May 29, 42–43.
7. WILLCOCK, G. (1990) Dept. of Care of the Elderly, Frenchay Hospital. Personal Communication.
8. The Guardian 1989 October issue.
9. PRESS, G. A., AMARAL, D. G. SQUIRE, L. R. (1989) Hippocampal Abnormalities in amnesic patients revealed by high resolution magnetic resonance imaging. *Nature*, **341**, 54–57.
10. HUBBARD, B. M., ANDERSON, J. M. (1981) A quantative study of cerebral atrophy in old age and senile dementia. *J. Neurol. Sc.* **50**, 135–145.
11. HUBBARD, B. M., ANDERSON, J. M. (1981) Age, senile dementia and ventricular enlargement. *J. Neuro. Neurosurg & Psych.* **44**, 631–635.
12. BRADSHAW, J. R., THOMSON, J. L. G., CAMPBELL, M. J. (1983) Computed tomography in the investigation of dementia. *Br. Med. J.* **286**, 277–280.

### ACKNOWLEDGEMENTS

My grateful thanks to the Dawn James Trust for the provision of an MRI Scanner to the 3 D.H.A.s in Bristol, and to my fellow Trustees for allowing me time on the machine for this study. Also to the staff in the Centre for their help and to Sally Alden for typing the manuscript.

**Figure 1**

Microscopic section of a case of Alzheimer's disease to show neurofibrillary tangles and senile plaques.

**Figure 2**

A coronal section of the brain of a patient with Alzheimer's disease. Note the severe shrinkage of the temporal lobe (left, compared with the normal brain, on the viewer's right).

**Figure 3**

A coronal slice through the anterior aspect of the temporal horn and the hippocampus. Inversion Recovery sequence. Normal control.

**Figure 4**

Coronal slice in the patient, thought clinically to be suffering from Alzheimer's disease. The ratio of temporal lobe area to hemisphere area was 1:5, and this was considered to be strong supporting evidence for the clinical diagnosis. The tracker-ball cursor lines are shown in position.

DEMENTIA

ALZHEIMER'S DISEASE

MULTI-INFARCTS — isolated; lacunar; periventricular lucencies

COMBINED

INTRACRANIAL LESIONS - tumours, subdurals, L.P.H.

TOXIC - alcohol, drugs (sedatives, tranquillisers, hypotensive etc.) infection

HUNTINGTON'S CHOREA. PARKINSON'S

HYPOTHYROIDISM. ANAEMIA. LIVER DISEASE.

..................

SENESCENT FORGETFULNESS

DEPRESSION

**Table 1**

**Short list of causes of dementia.**

## Figures and Tables

**Figure 1 f1:**
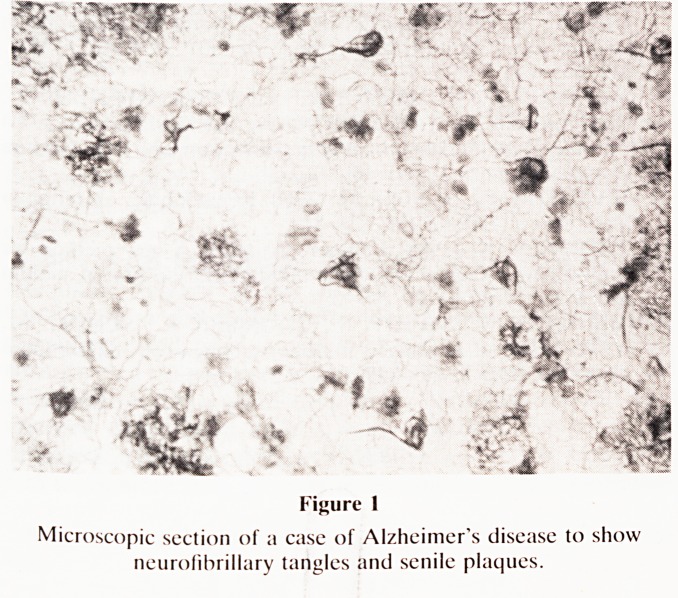


**Figure 2 f2:**
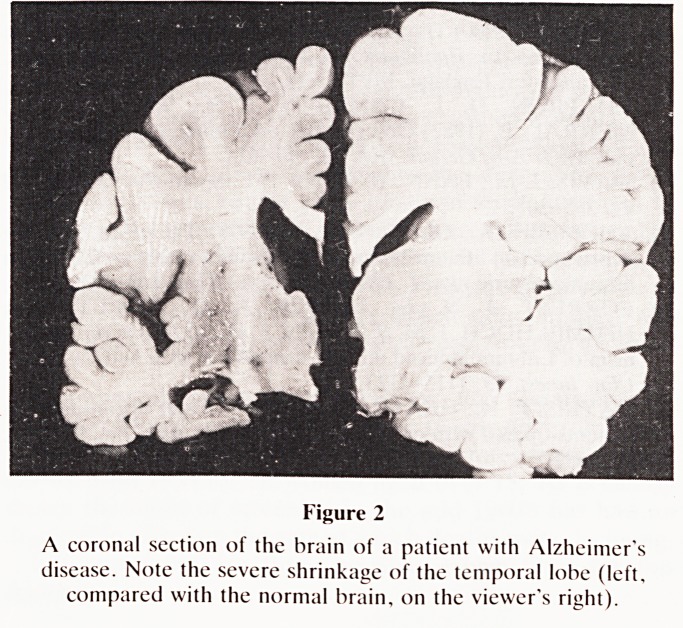


**Figure 3 f3:**
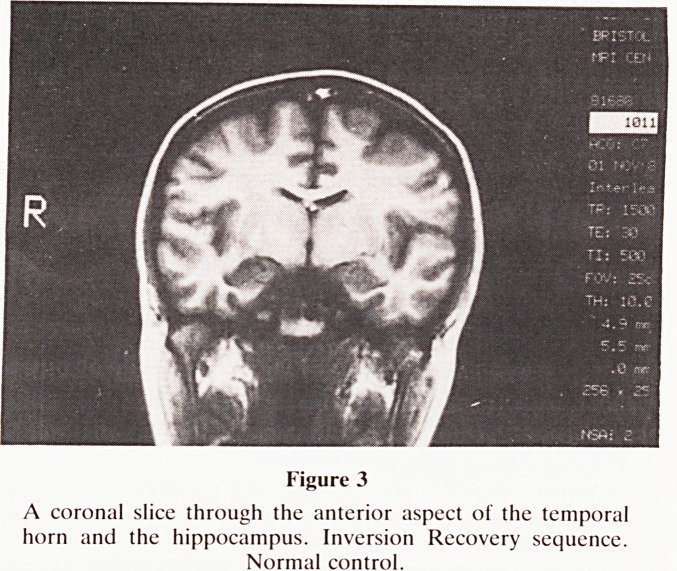


**Figure 4 f4:**